# 628. Characterizing a Sulbactam-Durlobactam Challenge Set of Acinetobacter baumannii Surveillance Isolates with Rapid Genotypic Testing (Carba-R) and Whole Genome Sequencing

**DOI:** 10.1093/ofid/ofaf695.195

**Published:** 2026-01-11

**Authors:** Tomefa E Asempa, David P Nicolau

**Affiliations:** Hartford Hospital, Hartford, CT; Hartford Hospital, Hartford, CT

## Abstract

**Background:**

*Acinetobacter baumannii* is emerging as an increasingly important opportunistic human pathogen. A 2023-2024 nationwide surveillance study focused on *A. baumannii-calcoaceticus* complex isolates among hospitalized patients (n=523) revealed that the majority of isolates were susceptible to sulbactam-durlobactam (SUD) (MIC_50/90_, 2/4 mg/L; 96.9% susceptible) with 3.1% of isolates (n=16) identified as non-susceptible. Comparative whole genome sequencing (WGS) and rapid genotypic analysis were performed to evaluate resistance mechanisms in this subset of non-susceptible isolates.

**Table 1.**
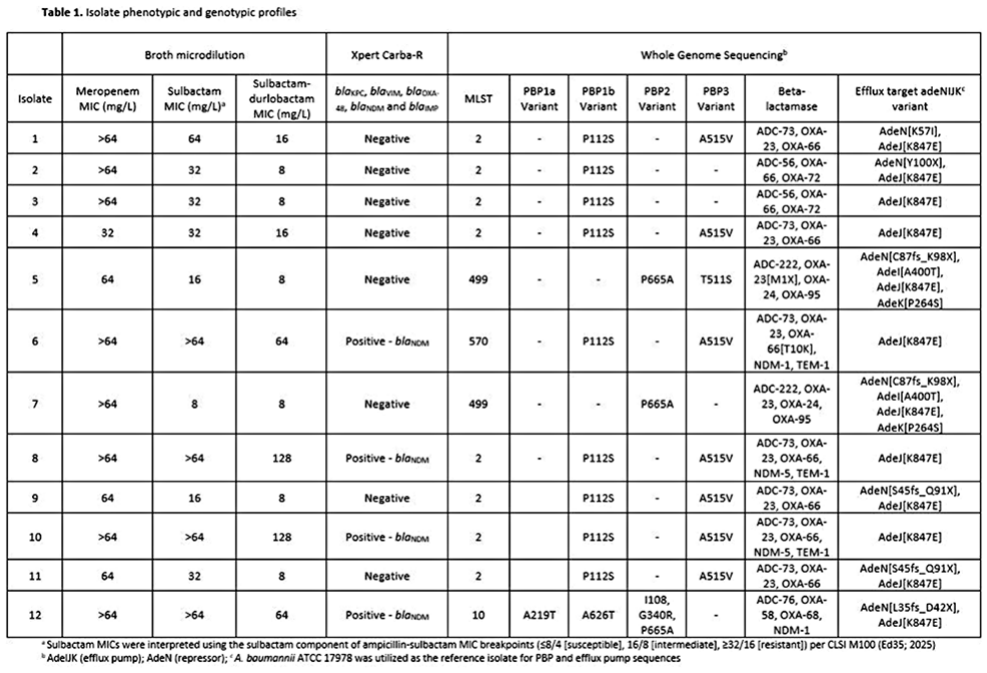
Isolate phenotypic and genotypic profiles

**Methods:**

Of the 16 non-susceptible isolates, 12 were available for genotypic testing from contributing hospitals. WGS was performed, and data were analyzed using open-source tools while Xpert CarbaR was performed according to manufacturer instructions to detect *bla_KPC_, bla_VIM_, bla_OXA48_, bla_NDM_* and *bla_IMP_* carbapenemase genes. Isolates were epidemiologically typed through multi-locus sequence typing using the Institute Pasteur scheme.

**Results:**

SUD non-susceptible isolates were all carbapenem-resistant with meropenem MICs of ≥8 mg/L (Table 1). The globally dominant clone ST2 was the most prevalent sequence type identified. Emerging clones ST10 and ST499 were also observed. All sequenced strains harbored genes for *Acinetobacter*-derived cephalosporinase (ADC) Ambler class C β-lactamase such as ADC-73, as well as various Ambler class D OXA β-lactamases (OXA-23, OXA-58, OXA-66 carbapenemases). A metallo-β-lactamase (NDM) was identified in 4 isolates. Target site alterations in penicillin-binding protein 3 (PBP3) and mutations in the AdeIJK efflux system and its transcriptional regulator AdeN were noted (Table 1). Carba-R concordance with WGS was noted, with identification of 4 *bla_NDM_*-positive isolates.

**Conclusion:**

All isolates exhibited mutations in efflux genes *adeNIJK* (which durlobactam is a substrate for) in addition to a mutation in PBP3 (n=8) or presence of an NDM gene (n=4). This study contributes to our understanding of clonality as well as the interplay of NDM production and mutations in efflux pumps and PBP present in SUD non-susceptible *A. baumannii* isolates. Further experiments are needed to identify the contribution of specific efflux gene variants to SUD non-susceptibility.

**Disclosures:**

All Authors: No reported disclosures

